# Differential Expression of Brain Cannabinoid Receptors between Repeatedly Stressed Males and Females may Play a Role in Age and Gender-Related Difference in Traumatic Brain Injury: Implications from Animal Studies

**DOI:** 10.3389/fneur.2014.00161

**Published:** 2014-08-28

**Authors:** Guoqiang Xing, Janis Carlton, Xiaolong Jiang, Jillian Wen, Min Jia, He Li

**Affiliations:** ^1^Department of Psychiatry, Uniformed Services University of the Health Sciences, Bethesda, MD, USA

**Keywords:** stress, anxiety, brain cannabinoid receptors, sex dimorphism, TBI outcome

## Abstract

Inconsistent gender differences in the outcome of TBI have been reported. The mechanism is unknown. In a recent male animal study, repeated stress followed by TBI had synergistic effects on brain gene expression and caused greater behavioral deficits. Because females are more likely to develop anxiety after stress and because anxiety is mediated by cannabinoid receptors (CBRs) (CB_1_ and CB_2_), there is a need to compare CB_1_ and CB_2_ expression in stressed males and females. CB_1_ and CB_2_ mRNA expression was determined in the amygdala, hippocampus, prefrontal cortex (PFC), and hypothalamus of adolescent male and female rats after 3 days of repeated tail-shock stress using qPCR. PFC CB_1_ and CB_2_ protein levels were determined using Western blot techniques. Both gender and stress had significant effects on brain CB_1_ mRNA expression levels. Overall, females showed significantly higher CB_1_ and CB_2_ mRNA levels in all brain regions than males (*p* < 0.01). Repeated stress reduced CB_1_ mRNA levels in the amygdala, hippocampus, and PFC (*p* < 0.01, each). A gender × stress interaction was found in CB_1_ mRNA level in the hippocampus (*p* < 0.05), hypothalamus (*p* < 0.01), and PFC (*p* < 0.01). Within-sex one-way ANOVA analysis showed decreased CB_1_ mRNA in the hippocampus, hypothalamus, and PFC of stressed females (*p* < 0.01, each) but increased CB_1_ mRNA levels in the hypothalamus of stressed males (*p* < 01). There was a gender and stress interaction in prefrontal CB_1_ receptor protein levels (*p* < 0.05), which were decreased in stressed females only (*p* < 0.05). Prefrontal CB_2_ protein levels were decreased in both male and female animals after repeated stress (*p* < 0.05, each). High basal levels of CBR expression in young naïve females could protect against TBI damage whereas stress-induced CBR deficits could predict a poor outcome of TBI in repeatedly stressed females. Further animal studies could help evaluate this possibility.

## Introduction

There is a growing body of literature supporting a gender effect on the acute response and long-term outcomes of TBI, yet the findings are inconsistent (1–5). Several studies suggest that gender differences in TBI outcome may be age-dependent. In a recent retrospective mortality study, involving 10,135 prepubescent (0–12 years), and 10,145 pubescent (12–18 years) hospitalized patients who sustained isolated moderate-to-severe TBI (defined as a head Abbreviated Injury Scale (AIS) score of 3 or greater). Ley et al. (6) found a significantly reduced mortality rate in prepubescent patients than in pubescent patients (5.2 vs. 8.6%, *p* < 0.0001). Additionally, females in the pubescent but not in the prepubescent age group showed a significantly greater decrease in mortality than males. Groswasser et al. (7) also reported a significantly better predicted-outcome for young females than for males under the age of 18 with comparable levels of TBI severity. Barr (8) reported that high school girls with TBI outperform boys of the same age on selected measures of processing speed and executive functions. Similar gender specific findings have been reported by others (9–12). However, other studies demonstrated that older women took significantly longer time than men to recover from TBI, after controlling for age, injury severity, mechanism of injury, and comorbidities (13–15). The mechanism for the inconsistent gender effect across different age groups is unknown.

Both genetic and epigenetic/environmental factors could be involved (16–19). Early stress exposure has been recognized as an important mechanism for neuropsychiatric disorders (20–22). Stress and stress-related anxiety could also influence TBI outcome as people who exhibited high levels of acute stress symptoms and anxiety had poor TBI outcome (23). A significant portion of the US military personnel returning from Iraq and Afghanistan battlefields have experienced persistent somatic pain, as well as comorbidity of mild traumatic brain injury (mTBI) and post-traumatic stress disorder (PTSD) (24–30). In a logistic regressions study of 2,348 veterans of Operation Enduring Freedom (OEF) and Operation Iraqi Freedom (OIF) (51% female), Iverson et al. (31) reported significant associations between probable TBI, symptomatic anxiety, and symptomatic physical health in both genders. Additionally, TBI is more strongly associated with all health symptoms for females and symptomatic anxiety and physical health for male veterans without probable PTSD (31). To examine the potential influence of repeated stress on the outcome of TBI, we recently reported that repeated stress followed by TBI had synergistic effects on the expression of brain mitochondrial electron transport chain complex subunits, and caused more severe behavioral deficits in male animals (females were not examined in that study) (32).

It is not clear if stress could have an equal influence on the outcome of TBI in males and females, although a greater impact of stress on the psychological outcome of females is well known. Our recent animal model studies of PTSD have shown that brain cannabinoid receptors (CBRs) are more rapidly depleted in the cerebella and brain stems of stressed female adolescent rats than in males (32, 33). Other studies suggest that endocannabinoids (eCBs) and CBR activity are involved in the functional recovery of animal experiencing repeated stress and TBI (32–37).

From the results of these studies and other evidence, we hypothesized that CBR-mediated activity may be a critical mechanism linking PTSD and TBI and is responsible for gender difference in PTSD and TBI. We are intent on investigating neuroprotective factors in male and female rats to evaluate how this may relate to recovery following TBI. While actual TBI procedure was not part of the current study design, the findings may translate to issues regarding TBI and co-occurring stress as evidenced in diagnoses such as PTSD.

Anandamide and 2-arachidonoylglycerol (2-AG) are the main components of brain eCBs. eCBs are synthesized upon demand through enzymatic cleavage of membrane lipid precursors and immediately released into the synaptic space. Anandamide has a higher affinity for the CB_1_ than for CB_2_ receptors (38), which are highly expressed in the hippocampus, striatum, cerebellum, and cortex (39). 2-AG has a low affinity for CB_1_ but is more abundant than anandamide (>200-fold) in the brain.

Endocannabinoids are synthesized upon demand through enzymatic cleavage of membrane lipid precursors and are immediately released into the synaptic space. They induce complex neuroprotective, anxiolytic, and modulator effects on brain structure and function via the activation of CBRs (mainly CB_1_ and CB_2_). Anandamide and 2-arachidonoylglycerol (2-AG) are the main brain eCBs and can alleviate blood–brain barrier dysfunction, brain edema, lesion volume, neuronal death, and improve behavioral performance in rodent models of TBI through multiple mechanisms (40–45). Anandamide has a higher affinity for CB_1_ receptors than for CB_2_ receptors (38), which are highly expressed in the hippocampus, striatum, cerebellum, and cortex (39). 2-AG, on the other hand, has a low affinity for CB_1_ but is more abundant than anandamide (by >200-fold) in the brain. The neuroprotective effects of eCBs in TBI could also be mediated by CB_1_ receptor activation, which can inhibit anxiety, stress response, and the retention of aversive memories (46). Animals lacking CB_1_ receptors show hypersensitivity to stressful stimuli, increased anxiety-like behaviors, and higher mortality (reduced lifespan) (47–50). CB_2_ receptors are primarily expressed in peripheral immune cells; however, recent studies show that they are also expressed in microglia, dendritic cells, brain endothelial cells, and the subgroups of neurons in several brain regions (51–55).

Evidence supporting a role of eCBs in TBI-induced injury and/or neuroprotection includes the significantly elevated levels of 2-AG following TBI (41). When administered to mice with TBI, 2-AG decreased brain edema, inflammation and infarct volume, and improved clinical recovery (42–44). 2-AG also suppressed inflammation, tumor necrosis factor-a (TNF-a), and reactive oxygen species (ROS) in LPS-stimulated macrophages and LPS-stimulated mice (56).

In this study, we examined CB_1_ and CB_2_ receptor expression after repeated tail-shock stress in the amygdala, hypothalamus, hippocampus, and prefrontal cortex (PFC) of adolescent male and female rats to determine how the base-line CBR can be affected by chronic stress. These brain regions play key roles in stress response and emotional memory. Adolescent animals were studied because they are more sensitive to stress than adult, a trait that could have a significant influence on disease development in adulthood (57–60). Furthermore, a gender difference in TBI outcome has been shown for pubescent animals, but not for prepubescent ones (6).

## Materials and Methods

### Animals

Male and female Sprague–Dawley rats (*n* = 16, each) (Taconic Farms, Germantown, NY, USA) weighing 120–150 g (5–6 weeks old) were used in this study. Animals of the same sex were housed two per cage and raised at room temperature (22 ± 2°C) on a 12 h light–dark schedule (lights on 1800 h). Animals had *ad libitum* access to food and water. All experimental procedures were approved by the Institutional Animal Care and Use Committee of the Uniformed Services University of the Health Sciences, and were carried out in accordance with the NIH Guidelines for the Care and Use of Laboratory Animals.

### Stress protocol

Animals were left undisturbed for 7-day after arrival. The stress procedure consisted of a 2-h per day session of immobilization and tail-shocks over three consecutive days as reported previously (61). In brief, half of the animals (eight per sex group) were restrained in individual Plexiglas tube and given 40 electric shocks (2 mA, 3 s duration) at varying intervals (140–180 s). The control animals were handled daily for the same time period but were not subjected to the immobilization and tail-shock stress procedures. All animals were returned to their home cages immediately after exposure to the stress or control conditions.

### Tissue dissection

Following the last stress session on day 3, both the control animals and the stressed animals were decapitated after light anesthesia with halothane. The brains were rapidly removed. A Vibratome (Technical Products International, St. Louis, MO, USA) was used to cut 1.6 mm-thick transverse slices containing the whole amygdala region (Bregma −3.60 to −2.00 mm) from tissue blocks. The basolateral complex, composed mainly of the lateral and basolateral nuclei, was dissected from this slice laterally, as outlined, by the white matter tract of the external capsule (corpus callosum) and medially by the white matter tract of the longitudinal association bundle. This transverse slice (Bregma −3.60 to −2.00 mm) also contained the hippocampal dentate gyrus and CA1–CA3 regions as well as part of the hypothalamus. The PFC was similarly dissected. All tissue samples were immediately stored in pre-cooled isopentane (−40°C).

### Reverse transcription and quantitative real-time PCR

Dissected brain tissue samples were homogenized and total RNA was extracted using an RNeasy kit (Qiagen, Germany) according to the manufacturer’s protocol. One microgram of total RNA was reverse transcribed into first-strand cDNA using the RETROscript reverse transcriptase kit (Ambion, TX, USA) according to the manufacturer’s recommendations.

Fifty nanograms of the reverse transcribed RNA from the RT-reaction was used as the template for quantitative real-time PCR reaction with a final PCR reaction volume of 25 μl and a final concentration of the 5′ and 3′ PCR primers at 100 nM each. CB1 (TTTCCCACTCATTGACGAGAC, GTGAGCCTTCCAGAGAATGT) and CB2 (AAAGCACACCAACATGTAGCC, GGAACCAGCATATGAGCAGAA) qPCR primers were designed using Primer3 software (MIT, MA, USA) with the size of amplified cDNA ranging between 90 and 150 bp (34). Quantification of CB_1_ and CB_2_ mRNA expression was performed (in triplicate) using a two-step PCR reaction procedure on an iQ5 Real-Time PCR System (BioRad, CA, USA) using the SYBR Green SuperMix (BioRad, CA, USA). After initial denaturation at 95°C for 3 min, 40 cycles of primer annealing and elongation were conducted at 60°C for 45 s, followed by denaturation at 95°C for 10 s. Fluorescent emission data were captured, and mRNA levels were quantified using the threshold cycle value (Ct).

Fold change in mRNA expression was calculated using the following equation: Fold = 2^(Ct control − Ct stress)^. To compensate for potential variations in input RNA amounts and the efficiency of reverse transcription, data for CB_1_ and CB_2_ mRNA of each sample were additionally normalized by reference to the data obtained from house keeping genes β-actin (GenBank accession no. X62085) determined from the same sample. The fold change in the compensated mRNA expression data was calculated using the equation: fold change = 2^−ΔΔCt^, where ΔCt = target gene Ct − housekeeping gene (β-actin) Ct, and ΔΔCt is ΔCt control − ΔCt stress (or fold change) = 2^(ΔCt control − ΔCt stress)^.

### Western blot

Prefrontal cortex tissues from the stressed and control animals were homogenized and sonicated for 40 s in the T-Per tissue lysis buffer for western blot analysis (Pierce, IL, USA). Amygdala, hypothalamus, and hippocampus tissue proteins were not examined due to the limited amount of these tissues that were dissected. Protein concentrations were determined using a Bradford assay (BioRad, CA, USA). Aliquots of 20 μg proteins were separated by electrophoresis on NuPage gels (10%) and transferred to a polyvinylidene difluoride membrane before being incubated with the primary antibodies of CB_1_, phosphorylated-CB_1_, glycosylated-CB_1_, and CB_2_, diluted at 1:500 each (Santa Cruz Biotechnologies, CA, USA). The membranes were rinsed in a 0.01 M Tris-buffered saline solution (pH 7.4) containing 0.1% Triton X-100 for 30 min, blocked in 5% non-fat dry milk for 30 min and incubated overnight at 4°C with the primary antibody in a Tris-buffered saline solution containing 3% non-fat dry milk. Membranes were washed three-times with the Tris-buffered saline solution and incubated overnight at 4°C with a horseradish peroxidase-conjugated secondary antibody in the Tris-buffered saline solution containing 3% non-fat dry milk. Immunoreactive bands were visualized using horseradish peroxidase-conjugated anti-rabbit antibodies in a 1:3000 ratio, and ECL Western blotting detection reagents (GE Healthcare Bio-Sciences Corp., Piscataway, NJ, USA). The western blots were captured with a digital camera and the intensities quantified with NIH Image 1.62.

### Statistics

Data regarding the effects of gender and stress on CB_1_ and CB_2_ receptors for individual brain regions were analyzed using two-way ANOVA analyses. Because of the significant gender and stress interactions found in brain CB_1_ receptor expression, within-sex one-way ANOVA analyses were also conducted. A *p*-value of <0.05 was considered statistically significant.

## Results

**Figure 1:**
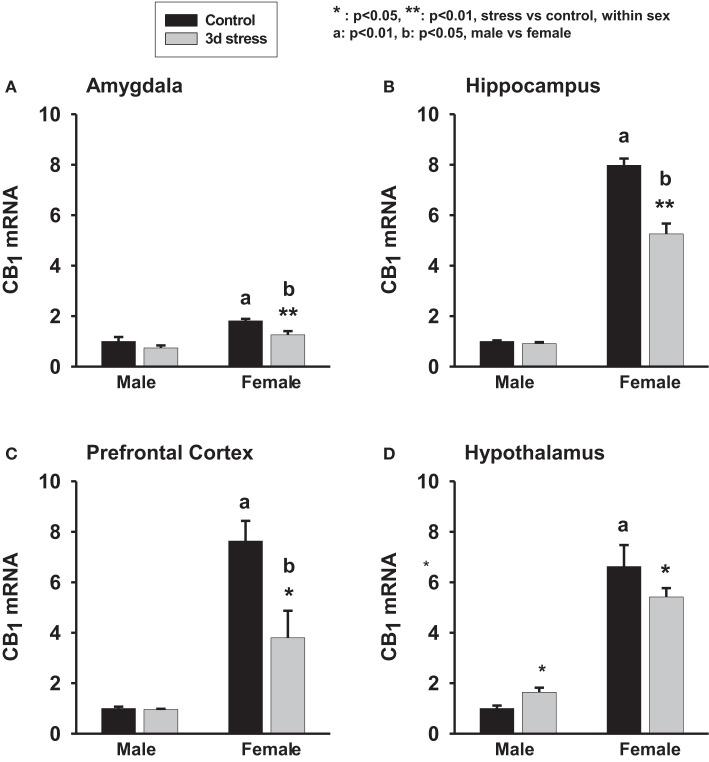
**Two-way ANOVA show significant effects of gender and stress**. Female adolescent rats show a greater baseline of CB_1_ mRNA expression in the amygdala, hippocampus, hypothalamus and prefrontal cortex than the males (*p* < 0.01, each). Three days repeated tail-shock stress significantly down-regulated CB_1_ mRNA levels in rat amygdala **(A)**, hippocampus **(B)**, prefrontal cortex **(C)** and hypothalamus **(D)**, especially in the female rats. Black column: control; gray column, stressed group, a, *p* < 0.01, male vs. female; b, *p* < 0.01: control group vs. stress group; *p < 0.05; **p < 0.01, control vs. stress within sex comparison.

Two-way ANOVA analyses revealed significant gender and stress effects on CB_1_ mRNA levels in the amygdala, hippocampus, and the PFC (*p* < 0.01, each). Overall, female animals exhibited higher basal levels of CB_1_ mRNA expression in the amygdala, hippocampus, and the PFC than male animals (*p* < 0.01, each) (Figure 1; Table 1). Stressed animals exhibited reduced CB_1_ mRNA levels in the amygdala, hippocampus, and the PFC when compared to those brain regions of the control animals (*p* < 0.01, each) (Figure 1). However, in the hypothalamus there was no significant difference between the CB_1_ mRNA levels in the stress and control groups (*p* > 0.05). A significant interaction between gender and stress on CB_1_ mRNA levels was found in the hippocampus (*p* < 0.05), hypothalamus (*p* < 0.01), and PFC (*p* < 0.01). Within-sex one-way ANOVA revealed decreased CB_1_ mRNA levels in the hippocampus, hypothalamus, and PFC of female animals (*p* < 0.01, each) but increased CB_1_ mRNA level in the hypothalamus of male animals after the stress (*p* < 0.05) (Figures 1A–D).

**Table 1:** **Relative fold change (mean ± SD) in CB_1_ and CB_2_ mRNA expression levels in the amygdala, hippocampus, prefrontal cortex (PFC) and hypothalamus of male and female adolescent rats after 3 days repeated (2 h/day) tail-shock stress**.

	Amygdala			Hippocampus			PFC			Hypothalamus		
	Baseline	3d Stress	Fold change	Baseline	3d Stress	Fold change	Baseline	3d Stress	Fold change	Baseline	3d Stress	Fold change
**CB_1_ mRNA**												
Male (*n* = 16)	1 ± 0.17	0.74 ± 0.1	0.74	1 ± 0.1	0.9 ± 0.1	0.9	1 ± 0.1	0.96 ± 0.03	0.96	1 ± 0.11	1.65 ± 0.18	1.65*
Female (*n* = 16)	1.8 ± 0.1	1.30 ± 0.14	0.69**	7.9 ± 0.26	5.3 ± 0.4	0.66**	7.6 ± 0.8	4.0 ± 1.1	0.5*	6.6 ± 0.8	5.4 ± 0.4	0.81*
**CB_2_ mRNA**												
Male (*n* = 16)	1.0 ± 0.19	1.1 ± 0.25	1.1	1 ± 0.2	0.69 ± 0.1	0.69	1 ± 0.4	0.8 ± 0.2	0.8	1 ± 0.1	1.5 ± 0.5	1.5
Female (*n* = 16)	1.7 ± 0.53	1.26 ± 0.37	0.59	2.5 ± 0.27	2.1 ± 0.75	0.83	3.4 ± 0.25	17.0 ± 8	4.9	6.0 ± 1.3	7.1 ± 0.8	1.2

The baseline mRNA level of control male group in each region was used as the arbitrary reference (=1) for the males and females. *p < 0.05; **p < 0.01, control vs. stress within sex comparison.

**Figure 2:**
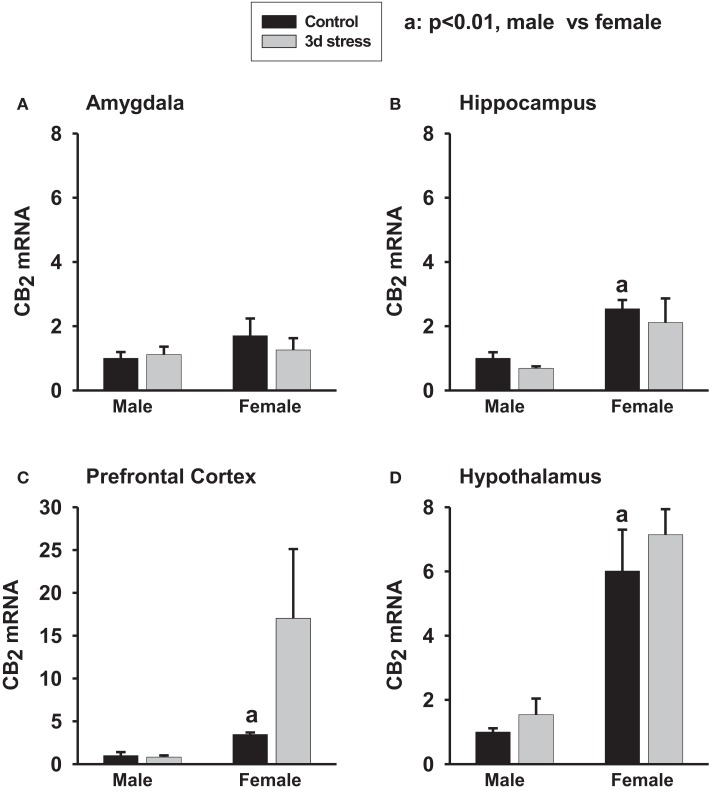
**Two-way ANOVA show that female rats exhibited greater CB_2_ mRNA expression in the amygdala (A) (*p* < 0.1), hippocampus (B) (*p* < 0.01), prefrontal cortex (C) (*p* < 0.01) and hypothalamus (D) (*p* < 0.01) than male rats**. Within-sex one-way ANOVA show that CB_2_ mRNA levels were significantly increased in the prefrontal cortex of female rats after the stress exposure (*p* < 0.05). Black column: control; gray column, stressed group, a, *p* < 0.01, male control vs. female control; *p < 0.05; **p < 0.01, control vs. stress within sex comparison.

Base-line CB_2_ mRNA levels were significantly higher in the hippocampus, hypothalamus, and PFC of female animals than in male animals (*p* < 0.01, each) (Figures 2A–D). CB_2_ mRNA levels, however, remained unchanged following stress.

**Figure 3:**
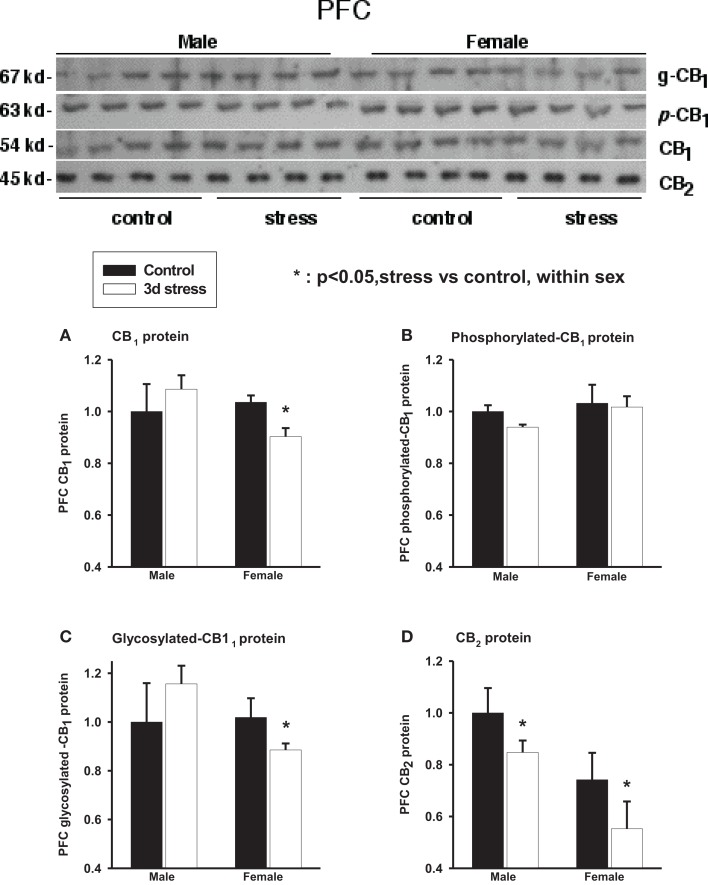
**Upper panel: representative western blots of total CB_1_ and CB_2_ receptor proteins; glycosylated (*g*-CB_1_) and phosphorylated (*p*-CB_1_) CB_1_ proteins in rat prefrontal cortex tissue homogenates**. Lower panel: two-way ANOVA of the western blot showing a trend level of stress × sex interaction in total CB_1_ proteins **(A)**; phosphorylated CB_1_ proteins (*p*-CB_1_) **(B)** and; glycosylated-CB_1_ proteins **(C)**. A within-sex one-way ANOVA showed significantly reduced total CB_1_ proteins and glycosylated-CB_1_ proteins in female prefrontal cortex (mean ± SD); CB_2_ protein levels were significantly reduced in the prefrontal cortex of both female and male rats after repeated stress **(D)**. The mean value of the male control group was used as the arbitrary reference = 1, *p < 0.05, control vs. stress within sex comparison, black column, control group; blank column, stressed group.

Two-way ANOVA analyses showed no significant gender or stress effects on total CB_1_ proteins, phosphorylated (p-CB_1_), or glycosylated-CB_1_ (g-CB_1_) proteins in the PFC (Figure 3; Table 2). There were, however, significant gender-by-stress interactions in total proteins and glycosylated-CB_1_ proteins (*p* < 0.05, each). Within-sex one-way ANOVA analyses showed significantly decreased total CB_1_ protein levels (*p* < 0.05) and glycosylated-CB_1_ protein levels (*p* < 0.05) in the PFC of stressed female rats but not in stressed males.

**Table 2:** **Relative fold change (mean ± SD) in CB_1_ and CB_2_ protein expression levels in the prefrontal cortex of male adolescent rats after 3 days repeated inescapable repeated tail-shock stress and female**.

	CB_1_ protein			p-CB_1_ protein			Glycosyl-CB_1_			CB_2_ protein		
	Baseline	3d Stress	Fold change	Baseline	3d Stress	Fold change	Baseline	3d Stress	Fold change	Baseline	3d Stress change	Fold change
Male (*n* = 16)	1 ± 0.11	1.09 ± 0.05	1.09	1 ± 0.03	0.94 ± 0.02	0.94	1 ± 0.16	1.16 ± 0.07	1.16	1 ± 0.1	0.85 ± 0.05	0.85*
Female (*n* = 16)	1.04 ± 0.03	0.9 ± 0.03	0.87*	1.03 ± 0.1	1.02 ± 0.05	0.98	1.02 ± 0.08	0.88 ± 0.05	0.87*	0.74 ± 0.1	0.55 ± 0.11	0.74*

The baseline CB protein value of the control male group was used as the arbitrary reference (=1) for the males and females. *p < 0.05, control vs. stress within sex comparison.

Two-way ANOVA analyses showed that prefrontal CB_2_ protein levels were greater in males than in females (*p* < 0.01) and were significantly suppressed in both sexes after repeated stress (*p* < 0.05). There was no significant gender-by-stress interaction in prefrontal CB_2_ protein levels.

## Discussion

A recent retrospective mortality study of TBI, involving more than 20,200 prepubescent and pubescent patients with moderate-to-severe TBI, showed that mortality rates were significantly lower in the prepubescent patients than in pubescent patients (*p* < 0.0001) (6). Within the pubescent group, it was further found that females had a significantly lower mortality rate than males. The mechanism underlying the age-dependent gender differences in TBI outcome is unknown. Besides a potential role of female sex hormones that may have protected the pubescent females, our studies as well as others suggest that brain CBR-mediated activity could play a critical role in the age-related gender difference in TBI outcome through at least two mechanisms: anxiolytic activity and neuroprotection.

In our study, female adolescent animals showed higher base-line CB_1_ and CB_2_ mRNA expression levels in the amygdala, hippocampus, hypothalamus, and PFC than the male adolescents (Figures 1 and 2; Table 1). That difference, however, disappeared rapidly after the repeated stress induced a larger reduction in CB_1_ mRNA levels in the female brain. Furthermore, although repeated stress down-regulated CB_1_ mRNA expression in the hypothalamus of female rats, caused the up-regulation of CB_1_ mRNA expression in the hypothalamus of male rats. This divergent result is consistent with the selective inhibition of hypothalamic neuronal activity by CB_1_ agonists in female but not in male guinea pigs (62), and the observation of a greater elevation of corticosterone in females than in males after stress (63). A reduction in CB_1_ protein and glycosylated-CB_1_ protein levels was also found in the PFC of the stressed female rats whereas a trend of increased CB_1_ protein was found in the male animals.

The higher base-line CB_1_ mRNA expression in the adolescent female rat brain when compared to their male counterparts is consistent with the reports of greater CB_1_ mRNA expression in the white blood cells of female humans (64, 65), higher eCBs content in the brains of female rats (66) and increased CB_1_ mRNA expression in the cerebella and brainstems of female rats (34). Because, CB_1_ activity is neuroprotective and a lack of CB_1_ activity in CB_1_ knockout animals is linked with increased mortality (67), our findings support the notion that greater base-line CB_1_ expression in female adolescent brains may underlie the reduced mortality in pubescent compared to the females with moderate-to-severe TBI when compared with adolescent males of the same TBI severity (6). While it is not yet known why such female-specific neuroprotection is present only in the pubescent but not in the pre- or post-pubescent populations, recent studies suggest that chronic stress when combined with high levels of stress hormone production but lower levels of female sex hormones production may deplete brain CBRs more rapidly, which could result in a large eCB/CBR deficit in the affected females.

To support this, Reich et al. (68) reported a lower level of CB_1_ expression in the hippocampus of socially isolated adult female rats than in their male counterparts. It should be noted that Reich et al.’s study differs from this study in many aspects including: (1) differences in stress paradigms (i.e., 3 days of repeated intense stress in our study vs. 3 weeks of chronic mild heterotypic stressors); (2) controls (naïve normal controls in this study vs. socially isolated controls); (3) feeding regimes (*ad libitum* feeding in this study vs. a frequent 14 h food/water deprivation); (4) housing environments (same sex pair-housing in this study vs. trio-housing with frequent wet cage rotation); (5) study times (acute phase of stress in this study vs. 3 weeks after chronic stress), and (6) hormone statuses (adolescent in our study vs. adult).

Increased crowding of unisex housing has been found to be stressful for female rats but anxiolytic for males and the opposite is true under isolated rearing (69). The unisex pair-housing in our study may be more stressful for the females than for the males and thus potentiating a greater loss of base-line CB_1_ receptors in the females after repeated stress. In contrast, chronic mild heterotypic stressors were more stressful for male rats but anxiolytic for females reared in isolation (69).

Gender-related differences in fasting-induced lipid catabolism also exist. It has been reported that females mobilize more fat reserve and thus catabolize more lipophilic eCBs than males during short-term fasting (70, 71). Animals in this study were fed *ad libitum* without fasting whereas the stressed animals in the Reich’s study experienced multiple episodes of food and water deprivation (>6 times in 14 h) that may have potentiated greater eCB release and CB_1_ receptor depletion in the stressed adult female brain of that study.

In this study, repeated stress caused a divergent pattern of prefrontal CB_1_ receptor expression between the males and females. Adolescent female rats displayed a significant reduction in prefrontal CB_1_ receptor expression. However, prefrontal CB_1_ receptor expression followed a positive trend in adolescent male rats, which became significant seven afterwards (33), reinforcing the findings of the divergent CB_1_ gene expression patterns after stress. This increased CB1_1_ expression in male PFC is consistent with the increased mitochondrial electron transport chain complex subunit expression in the PFC of stressed male animals (32). Because of the known anxiolytic and analgesic effects of eCBs and CB1_1_ activation, the more-rapid loss of CB_1_ in stressed adolescent female brains is consistent with the clinical observations of a greater prevalence and higher severity of anxiety symptoms such as increased sensitivity to fear signals, emotional disturbance, and pain in females after chronic stress exposure (72–76).

Ley et al. (6) showed that human prepubescent, regardless of sex, are better protected against TBI-caused mortality than human pubescent (and possibly the adults as well). Although the mechanism is unknown, developmental studies have shown that the level of CB1_1_ expression in the human PFC is highest after birth but declines rapidly during the postnatal and prepubescent periods and with age (77). Thus, potentially high levels of CB1_1_ expression and activity during the prepubescent periods of development may have provided equally strong neuroprotection against TBI-induced brain damage and mortality in both naive prepubescent males and females (6). While developmentally regulated decline in brain CB1_1_ expression and CB1_1_-associated neuroprotection may be partially compensated by increased sex hormone in naïve pubescent and young adult females, this compensation may be adversely affected in stressed females.

The mechanisms for the poor reported long-term poor outcome of TBI in the older female population could be more complex (78). Again, deficient brain CB_1_ activity, due to chronic stress, elevated stress hormone levels, and reduced sex hormone levels could all play a role in the female brain, leaving it more vulnerable to TBI damage.

It is possible that the neuroprotective effects of sex steroids in TBI (79) may act partially by upregulating brain eCB activity and CB receptor expression (77). Sex hormones during the estrous cycle have been linked with brain CB_1_ receptor density, which is reduced in the limbic forebrain and hypothalamus after ovariectomy and castration but can be restored after estradiol, progesterone, and testosterone administration in intact and ovariectomized/castrated rats (80–84). High levels of stress-induced corticosteroid secretion and base-line corticosteroids as well as slow clearance of corticosteroids could lead to reduced CB_1_ receptor levels in stressed females (85, 86). Chronic exposure to high level of corticosterone, CB_1_ agonists, and cannabinoids have been reported to downregulate CB_1_ receptor density, CB_1_ receptor binding, and CB_1_ mRNA expression in various brain regions of male and female animals (87–91).

It is noted that although a reduction in prefrontal CB_1_ and CB_2_ mRNA expression was not immediately observed in the male animals after 3 days of repeated stress, the expression was significantly decreased in the stressed male animals 7 days following the stress (33), suggesting a delayed pattern of CBR reduction in male adolescents after repeated stress when compared to the females. Other studies showed that 10 days of mild chronic stress (30 min restraint stress per day) upregulated CB_1_ binding in the PFC of adolescent and adult male rats that was resolved after 40 days recovery period. Furthermore, adolescents exposed to stress were found to have a sustained downregulation of prefrontocortical CB_1_ receptors in adulthood (92).

CB_1_ receptor activation in the forebrain and amygdala is anxiolytic (46, 93, 94). The loss of CB_1_-mediated anxiolytic and neuroprotective activity in these brain regions of both female and males could predict enhanced amygdala-mediated fear memory, especially in the females due to a greater propensity for CB_1_ reduction. Indeed, loss or inhibition of CB_1_ receptors in the amygdala, hippocampus, and PFC have been associated with the impaired ability to extinguish fear memories (50, 95, 96). PFC is known to exert a powerful inhibitory effect on amygdala activity and on fear extinction (97, 98) and it has been observed that the amygdala and hippocampus interact to mediate emotional memories (99).

Stress-induced reduction of brain CB_1_ and CB_2_ protein expression may also contribute to a more vulnerable brain structure and function (100–111) through multiple mechanisms in response to TBI, including increased microglia activation, inflammation and apoptosis, impaired blood–brain barrier integrity, compromised neuroprotection, and neuroregeneration in response to TBI (42, 112, 113). Activation of CB_1_ and CB_2_ receptors may minimize brain damage and promote tissue repair after TBI through the attenuation of injury-stimulated inducible nitric oxide (iNOS) and ROS in microglia (114), promoting neural progenitor (NP) proliferation, and neurosphere generation (115–118). These actions are abrogated when there is a deficit of brain CB_1_ receptors (119–121). Altered expression levels of brain and peripheral CB_1_ and CB_2_ receptors could also underlie changes in energy metabolism and body weight loss, both of which are common phenomena resulting from TBI, due to their direct influence on feeding, glucose uptake, fatty acid synthesis and triglyceride accumulation, energy expenditure, and metabolic homeostasis (122–127).

It is tempting to speculate that brain CBR deficit, associated with stress, age, gender, and anxiety/agitation, could play a central role in the individual variations in the outcome of TBI (128). While TBI is not part of the current study, the findings of stress-induced CBR deficits may translate to issues regarding TBI and co-occurring stress as evidenced in diagnoses of comorbidity of PTSD and mTBI in military personnel returned from Iraq and Afghanistan war zone that could advance our understanding of the neuroprotection of the consequences of TBI. Strategies to reduce gender and stress-related brain CBR deficit and agents to restore CBR activity could become potentially effective therapies for TBI. Indeed, treatment with synthetic 2-AG resulted in attenuated edema formation, infarct volume, and blood–brain barrier permeability in a mouse model of TBI, an effect dose-dependently attenuated by a CB_1_ antagonist (41). And partial inhibition of 2-AG degradation, improved motor coordination, and working memory performance in mice model of TBI (37). Selective and highly potent cannabinoid CB_1_ and CB_2_ receptor agonist showed a pronounced neuroprotective effect in a rat TBI model (129). A potent and CB1 and CB2 receptor agonist, when applied before, during, and after transient occlusion of the middle cerebral artery, significantly and dose-dependently reduced cortical lesion sizes and motor deficits (130).

In summary, we found a higher basal value of CBRs in the forebrain of adolescent female animals, which was significantly reduced after repeated stress. Because of the known anxiolytic and neuroprotective effect of eCB and CBR activities, this high base-line CBR may provide a neuroprotective mechanism for the improved outcome of prepubescent and pubescent females with TBI. The stress-induced reduction of CBR may underlie the poor long-term outcome of older female TBI patients who may also be experiencing postmenopause-related reductions in reproductive hormones. As brain eCBs and CBR activity is implicated in age and gender-dependent difference in the outcome of TBI and PTSD, further studies in this direction are required.

## Conflict of Interest Statement

The Guest Associate Editor Yumin Zhang declares that, despite being affiliated to the same institution as authors Guoqiang Xing, Janis Carlton, Xiaolong Jiang, Jillian Wen, Min Jia and He Li, the review process was handled objectively and no conflict of interest exists. The authors declare that the research was conducted in the absence of any commercial or financial relationships that could be construed as a potential conflict of interest.
